# Ricoeur’s hermeneutic arc and the “narrative turn” in the ethics of care

**DOI:** 10.1007/s11019-021-10020-9

**Published:** 2021-04-29

**Authors:** Maria Teresa Russo

**Affiliations:** Chair of Bioethics and Moral Philosophy, Faculty of Education, Roma Tre University, Rome, Italy

**Keywords:** Hermeneutics, Medical ethics, Medical humanities, Narrative medicine, Nursing, Ricoeur Paul

## Abstract

“Patient-centred care” is the recent response to the malaise produced in the field of health care from the point of view both of a technical mentality and the paternalistic model. The interest in the story-telling approach shown by both the humanities and the social sciences has favoured a “narrative turn” in medicine too, where the new ethics of therapeutic relationship consider the hermeneutic method a means by which to integrate evidence and subjectivity, scientific data and patient experience. The aim of this paper is to show how Ricoeur’s theory of “threefold mimesis” makes a conceptual contribution to the use of narrative interviews in nursing and also be successfully transferred into and applied in the field of healthcare in general. First, the paper examines how this narrative approach might open up new possibilities for the acquisition of in-depth knowledge of patients’ life experiences, a condition indispensable for the improvement of the quality of care. Secondly, it highlights how this Ricoeurian method seems capable of provide an opportunity for healthcare professionals to review their own understanding of the caregiver-patient therapeutic relationship, beginning with their confrontation with the patient’s world as revealed by the narrative they provide.

## Introduction

For some years now, in countries where technological progress has advanced rapidly, a feeling of unease has arisen following the application to medicine of the mechanistic paradigm, which has transformed the art of healing into a science based on *quantitative* data and *measurable* amounts. The inadequacy of the so-called Evidence Based Medicine (EBM) paradigm has been denounced by many of those seeking a model which, while remaining scientifically rigorous, brings together the evidence of data, the uniformity of protocols as well as the attention due to the individuality of the sick and their experiences. While undoubtedly useful, this model excluded other forms of rationality from clinical care, proving, in the long run, incapable of grasping the complexity of medical action and the multidimensionality of the reality of disease. The main criticism levelled against EBM is its simplistic approach to scientific knowledge, which prioritizes internal validity when including studies in clinical guidelines. Internal validity is a useful criterion during the discovery and corroboration phases, but it is inadequate when it comes to implementation, because health and healthcare, as non-linear phenomena emerging from their different components and clinical decisions, need to be fine-tuned to accommodate the individual needs and circumstances of patients (Fernandez et al. 2015).^1^

Hence the need to integrate EBM with a new scientific paradigm based on a broader concept of evidence and the concept of care,informed by practical wisdom—Aristotele’s *phronesis-*, which involves prudential assessment and critical attention to ethical issues regarding the effective human status of patients (Edmondson and Pearce 2007, 241). The introduction of a Taylorist kind of mentality, inspired by quantitative measurements and parameters of efficiency inspired by a “piece-time” view of healthcare, while it undoubtedly shortened intervention time in cases of urgency and reduced iatrogenic pathologies, prevented patients from being considered global, complex, holistic units, with personal and family histories, which needed to be interpreted and deciphered to some extent. Richard Sennett noticed (Sennett 2008, 48–49) that studies conducted on the models adopted in Western European healthcare systems revealed feelings of profound frustration among medical and nursing staff, due to the pressure of having to adapt to institutional parameters. He cited the example of Great Britain, where a “Fordist” healthcare model was adopted to cope with the problems caused by the deterioration of the system, based on monitoring the time that doctors and/or nurses dedicated to each patient.^2^ While this approach had improved the organisation of a series of processes from a quantitative point of view, it had also produced a marked deterioration in the quality of care. The problem was that the rational analysis of processes and their breakdown into programmable and controllable sequences had not adapted well to the delivery of care, which had as its object a reality, that of bodies, impossible to collocate totally within a system of classifications.^3^

The new ethic of therapeutic relationships, based on the so-called “patient-centred care”, has, therefore, reassessed subjective aspects, also as a value relating to the healthcare worker and patient alike, by attributing an interpretative-dialogical character to clinical practice. It also aimed at correcting the paternalistic paradigm, in an effort to turn patients into partners and involve them more and more in the decisions regarding their care, in line with the “no decision about me, without me” model (Coulter 2011). Slogans like “the patient as a partner” or “the patient first” are becoming increasingly more significant within the ambit of clinical relationships (Hutchinson 2017), although the technocratic mentality to which exclusive efficacy is often attributed, still survives in many areas.

The interest in storytelling and narrativity which emerged in the humanities and the social sciences has given rise to a “narrative turn” in medicine, where attention towards the hermeneutic method, aimed at integrating evidence and subjectivity, scientific data and patient experiences, has increased (Frank 1995; Charon 2007, 1265–1267; Charon 2008; Hurwitz and Charon 2013, 1886–1887). An essential contribution to change is also played by so-called Medical Humanities, thanks to the supplementary roles of art and literature, but, and above all, of philosophy, although there has been no lack of criticism of this narrative turn (Strawson 2004, 428–452).

In this context, the function of the nurse acquires particular significance, both in view of the effectiveness of the services provided and the real care of the patient, since nurses share with their patients and their patients’ families a narrative of the disease which is much broader in scope than any hastily compiled anamnesis (Benner and Wrubel 1989). As some scholars have observed, the nurse, by relating to patients and listening to their stories, can grasp, almost accidentally, useful clues about the disease which may not have been included in a diagnostic checklist. As Patricia Benner (1984, XXV) observes “The nurse-patient relationship is not a uniform, professionalized blueprint but rather a kaleidoscope of intimacy and distance in some of the most dramatic, poignant, and mundane moments of life”.

Sennett also reports that in the “old” health service, because of a consolidated kind of practice, nurses, in addition to carrying out their duties, often stayed in the ward to listen to the stories of elderly patients, although this was not a part of their remit. Thanks, however, to these informal moments they were able to glean clues useful for diagnosis or therapy, proof that activities of assistance and care require a more global type of attention located within “a liminal zone between problem solving and problem finding” (2008, 48). Havi Carel emphasises the ethical relevance of taking into account the patient’s narrative and point of view in a relationship of care. Carel applies Miranda Fricker’s thinking to the healthcare sector (Fricker 2007). Fricker defines as *epistemic injustice* the denigration or downgrading of someone’s testimony due to listener prejudice, something that is tantamount to oppression. In a relationship with patients, this type of injustice can actually occur. When patient credibility is denied, he/she is also offended as a cognisant subject*.* This author distinguishes two types of epistemic injustice of which patients can be victims in clinical practice: *testimonial injustice* and *hermeneutical injustice*. The first occurs when the patient’s narratives are excluded from epistemic consideration because they are considered suspicious or cognitively unreliable and dismissed by doctors as irrelevant because of being confused, overly emotional or seen as a useless waste of time. The self-censorship of the patient who disqualifies his own testimony falls within this type of epistemic injustice (Carel and Kidd 2014, 529–540). Conversely, hermeneutical injustice takes place when patients are unable to articulate their own experience of illness in a conceptually clear way, which is why it is not acknowledged as an important source of information but subjected to the authority of physicians. These two kinds of injustice are frequently met by patients in clinical practice when they try to express their experiences and perplexities regarding the treatment of their illnesses. Carel argues that epistemic justice, on the contrary, requires recognition of the testimonies and narrations of sick people and the attribution to them of epistemic consideration, therapeutic and diagnostic value. Healthcare professionals enjoy an epistemic privilege by virtue of their training and skills. Indeed, both healthcare workers and the sick are epistemically privileged, but in the doctor-patient relationship a sort of implicit hierarchy emerges where only the epistemic state of the health professional “really matters”, to the detriment of that of the patient. In her opinion, the practices of the contemporary healthcare system foster epistemic injustice by preferring certain styles of well-articulated narratives, articulated if possible in correct medical jargon and favouring impersonal relationships, so that the patients, she sustains, are seen far too often as the “objects” of diagnostic practices, rather than as active participants in the therapeutic process.

For these reasons, from the 1970s onwards, the methodological literature of the health sciences has made increasingly more frequent reference to hermeneutics, inspired initially by Kierkegaard and Heidegger (Koch 1995, 827–836), Gadamer, in particular, and, more recently, by Paul Ricoeur (Charalambous et al. 2008, 637–642). The contribution made by hermeneutics has even favoured a redefinition of the aims and nature of the health sciences as a response to the dissatisfaction researchers have expressed concerning the frequently dominant positivist and Cartesian approaches. This method has been applied mainly in nursing, a field more attentive to the true meaning of the experiences of patients, as an alternative paradigm upon which to base new qualitative research.

It is the literature of nursing which proves most sensitive when it came to registering the need for a paradigmatic shift, precisely because direct contact with patients and their experiences leads to consideration of nursing as a human science focused on people, where the notion of evidence was derived mainly from practice itself (Rolfe 2015, 142–152). It is to be hoped that this sensitivity will extend to the whole field of medical practice, so as not to create a dangerous dichotomy: that between an increasingly distant medicine and a nursing care that seeks proximity to the patient.

This paper will explore the theory of “Threefold Mimesis” developed by Ricoeur to illustrate how it has been used in the nursing field when interviewing patients. It will point out that this method of narrative analysis proves useful to the elaboration of a clinical case history which truly reflects the situation of a patient and to the consequent establishment of an effective care relationship, not only in nursing field, but also in medical practice. Finally, this article will show how the third level of Mimesis, that is, “refiguration”, can constitute a resource for healthcare personnel enabling them to reflect on critically the ethical quality of their care relationships.

## Hermeneutics as a methodology of renewed healthcare ethics

While Gadamer (2004) provided a general theory of human understanding, Ricoeur’s contribution may be valued above all in relation to the key question of the hermeneutic circle of explanation and understanding, aimed at bridging the gap between ontological and critical hermeneutics, an aspect explored less, it would seem, by the German philosopher.

The perspective that has been adopted by nursing literature dedicated to the new frontiers of care ethics, is that of hermeneutic phenomenology, often referred to as “interpretive phenomenology” (Chan et al. 2010) because it aims at combining the simple “knowledge” of facts with an “understanding” of meanings.^4^ The three main targets of the criticism advanced are the atomistic individualism of the subject isolated from others, the positivist model of the patient seen as a simple organism and the Cartesian conception of the disembodied and rationally self-sufficient being: all three visions, set within a linguistic and cultural tradition, impede a global, holistic understanding of individual patients in their singularity and in the complexity of their social relations. The goal is ultimately the rejection of what has been called *disengaged care* (Chan et al. 2010), where clinicians avoid involving themselves from a personal point of view in the singularity of the patient, by generalising the personal experiences of the sick.

From this perspective, it is acknowledged that hermeneutic philosophy plays an important role in nursing, and it is possible to go so far as to speak of the “hermeneutic conception” of nursing care (Frechette and Carnevale 2020). For some authors, “introducing the hermeneutic dimension into caring science implies that language, metaphors, words, concepts, and texts are given a central place in the formation of knowledge” (Eriksson 2002, 62). Therefore, hermeneutics provides a new view of care, seen as more than mere methodology, so much so that the term “nursing science” is replaced by “caring science” and endowed with exquisitely humanistic substance aimed at catering for the person considered in all his /her entirety and inspired by an ethic of compassion, where ethics precede ontology.^5^

This view favours a new approach to clinical history and underlines the specificity of nursing. From a medical point of view, clinical history is different from the reality of nursing. The former focuses on the disease while the latter foregrounds disease-management aimed at alleviating pain and discomfort and creating favourable healing conditions. There is a thin line between the clinical histories of physicians and nurses: although similar methods of investigation are used and both have disease as their starting point, the latter aims at promoting a more diversified conception of health (Dillon 2007). Knowledge of the patient is essential, actually, when seeking to identify and promote, as far as possible, the level of health the patient deems desirable.

In particular, this kind of knowledge fulfils the function of support of the four orientational pillars of clinical history and the practice of care: orientation towards care, orientation towards narrative, orientation towards time and orientation towards the body (Dillon 2007).

Care is performed professionally thanks to the encounter made possible through the medium of the body. To bring a glass of water to those who ask for it is a generally comprehensible gesture of care, while recognising signs of dehydration while bringing it is concern at a different level. Nurses can be truly touched by their patients’ lives only if they are prepared to be affected, while it is the body that provides the opportunity of being touched and met. If not, in keeping with Heidegger’s comparison (Heidegger 1962, 81), one would simply stand next to another, like a chair leaning up against a wall, whose simple contiguity cannot be compared to that of contact between a hand and a wall. Nurses can meet the other only if they are capable of creating conditions of authentic encounter, laying down the protective armour of their emotional disengagement and opening up to the world of the other which is made of fragility and suffering.

To achieve truly *engaged caregiving*, the traditional anamnesis needs to be transformed into a semi-structured interview aimed at getting to know the other person, thanks to hermeneutic investigation. In this case, the list of questions regarding both past and current health concerns, is expanded as the result of broader interest in the comparative meaning of important details regarding the living world of the patient and aimed at achieving better mutual understanding. The use of Gadamerian and Ricoeurian hermeneutic teaching actually permits us to grasp the process which from self-interpretation leads to a “fusion of horizons” (Gadamer 2004) between nurse and patient, essential to the construction of a bridge between their reciprocal horizons of meaning and the achievement of understanding.

Orientation towards narrative makes it possible to avoid focusing on the individual episodes observed or reported by the patient but permits their collocation within the broader horizons of a complete story. With regard to this it is significant that the Ricoeurian idea whereby history is a two-dimensional narrative which permits us to understand it. One dimension regards a chronological sequence of episodes, the other the construction of a meaningful whole (Ricoeur 1984, 150–151). If events are to acquire importance only thanks to their configuration within a narrative, that is, in the structuring of a plot, which is no simple serial enumeration of facts, but their organisation “in an intelligible whole” (Ricoeur 1984, 110), it is necessary that a clinical history not be limited to a collection of isolated data regarding a scattered dissemination of events—vaccinations, past operations, allergies, etc.,—as was the tradition, but that it reconstruct the story of a patient’s health in its entirety.

The organisation of a narratively-arranged clinical history can make use of different strategies: from the initial ritual question regarding the reasons for the medical visit, one can enlarge by asking open questions—“Can you tell me more? what does this mean for you? What do you think contributed to this situation?”- to encourage the patient to tell his/her story by soliciting and reinforcing listening and attention through non-verbal acts like a nod of the head and/or prolonged eye contact.

Understanding patients’ situations also requires paying attention to the temporal horizons of their experiences, where past, present, and future never exist independently, but involve and merge with each other. Referring to Heidegger and Gadamer, the literature of nursing underlines the fact that the medical model, whereby time is considered only within clinical history, that is, according to the evolutionary progress of the disease and which, even if effective, is insufficient in any case, because it does not allow for a reading and interpretation of the meaning that health assumes from time to time during the lifetimes of patients. While traditional clinical history is disease-centred, a “history of health” could lead to greater understanding patients’ actual conditions, including their preparation for treatment. This approach, where the hermeneutic teaching of Ricoeur is applied to the historicity of the experience of health—the correlative of the historicity of the person- provides for interpretations of the different meanings attributed to health as experienced in the past, the present, and the future by the person receiving care. This means that it is mandatory not to consider events in isolation but as aspects of an interweave permitting the detection of meaningful details which, otherwise, may have remained implicit or hidden. The history of a person’s health changes over time as the horizons of meaning evolve little by little and every single event is seen against an ever-shifting backdrop. Furthermore, the weight of culture and tradition should not be underestimated, as well as what Taylor calls “social imaginaries” (Taylor 2004),^6^ which form the complex background of knowledge and practices within which we move and which, by influencing our self-interpretation, modify our expectations and interpersonal relationships. In the encounter between with the patient what we might call an instance of the “hermeneutics of the body” assumes particular importance capable of permitting one to overcome the initial opacity of the relationship or paying simple attention to symptoms. It is a matter of going beyond the “physical body”, to understand it, rather, as a “bearer of meaning”, whose signals need to be intercepted and interpreted. From this perspective, the sight and touch, in particular, can often deliver much more than verbal language, by providing a vast range of fundamental elements essential to a true understanding of patients’ experiences. It is a matter of going beyond the “physical body”, to understand it instead as a “bearer of meaning” whose signals need to be intercepted and interpreted. In this perspective, the sight and especially touch can offer, much more than verbal language, a vast range of fundamental elements for understanding patients’ experiences.

## Ricoeur’s *Threefold Mimesis* as a method for the interpretation of patient narratives

The adoption of the Ricoeurian method has acted, in part, as a turning point in the way the function of hermeneutics can be understood in the literature of nursing care, which had as its first source of inspiration, if we exclude references to Heidegger, the implications of the four key concepts Gadamer presented in his *Truth and Method* (2004): prejudice, fusion of horizons, the hermeneutic circle, and play (Austgard 2012, 829–834).

In Ricoeur we find a clearer approach to the relationship between ontology (the interpreter) and epistemology (the interpretation) in his *Threefold Mimesis*, a key by which to access the significance of lived experience as well as different approaches to oral narrations and written texts.

In the literature of nursing, the attention paid to Ricoeurian hermeneutics is regards above all processes of interpretation, seen as amalgamation into a single multidimensional act of explanation and understanding applied to the narratives of patients and healthcare professionals which, on the one hand, foster a better administration of care, on the other, a practice of care informed more by awareness of abilities and limitations. For a kind of nursing practice centred on patients’ expectations, the Ricoeurian idea appears particularly effective when, at the end of the explanatory pathway, the understanding of a narrative not only reveals aspects of the patients’ worlds, but also permits the subject—in this case the nurses- to review their understanding of themselves starting from a confrontation with that world revealed by the narrative.

Ricoeur’s theory of interpretation is used, therefore, as a method of textual analysis applied to patients’ narratives, which proceeds through a number of different steps: from distancing to appropriation, from explanation to understanding, up to the point of hypothesis and validation (Ricoeur 1991, 142; 2016, 107–126). This way, while lived experiences remain private and non-transferable, their meanings become public because they provide a more complete and deeper understanding of the phenomena of illness and treatment. The application of the method theorised by Ricoeur appears particularly in line with the theoretical models of nursing care, whose pivot is a view of human beings in constant interaction with their environment, seen as a set of conditions and external influences capable of preventing, curing or contributing to the evolution of the disease experienced in a personal and non-transferable manner. Charalambous believes that there are several reasons for considering the Ricoeurian dialectic as a non-dichotomy between explaining and understanding particularly suited to nursing care which many authors consider not so much an “objective science with one universal truth” as a practice where scientific and humanistic knowledge combine (Charalambous et al. 2008, 640). First of all, it abandons that brand of Cartesian dualism which separates the physical from the mental, the subjective from the objective, the observer from the observed to recover the holistic, circular perspective which characterises nursing theory. Secondly, the theory of interpretation, which acts as a hub between language and lived experience, is particularly effective for analyses of narratives and interviews, also when it comes to the consideration assigned to values, beliefs and cultures, elements fundamental to the understanding of the experiences of both researchers and participants. Finally, the co-implication of the interpreter with the object interpreted makes it possible not to place oneself outside of the investigation, but permits one to obtain a projection on the quality of one’s mode of care, in order to enhance it with new meanings, re-shape it, possibly modify it. The comparison with what a text can reveal of its own mode of being, turns the interpretative act into an occasion for a more authentic kind of self-understanding.

The theory of the *Threefold Mimesis* (Ricoeur 1984, 52 and ff) has been applied variously to nursing contexts, always in view of an ethos of care more attentive to the singularity and complexity of the sick person. Birthe D. Pedersen has devised a three-level analytical model inspired by Ricoeur’s theory which has constituted an interpretative tool for interviews with patients and observations in hospital (Pedersen et al. 2009, 6–73): naïve reading, structural analysis, critical interpretation, and discussion. This process requires movement from explanation to understanding and vice versa and allowance for the intrinsic validation of initial preconceptions and preconceptions.

These three levels refer to mainly the three moments of narrative theory, the so-called *three mimesis*, which are prefiguration (spontaneous and immediate relationship with the emotionally-experienced world), configuration (narrative construction and temporally organised narrative), and refiguration (return to the world of acting and of suffering, strengthened by previous experience and a new understanding of the world which have an impact at ethical level) (Ricoeur 1984, 52).

*Mimesis* is not an imitation of reality, but, in the Aristotelian sense it is intended as the process by which narratives acquire full intelligibility: not a copy or re-creation of the original, but its revelation and organisation over time, obtained by decoding its traces.

*Mimesis I* refers to life as lived before being formulated into spoken or written narratives, corresponding to Husserl’s world-of-life, that is, the daily, pre-scientific world, whose content is constituted by existential experiences like hope, fear, suffering. At this stage, the essential goal of a humanistic kind of health science is to bring to the fore elements that are important for the patients but which fail to be brought to the surface by means of objective scientific methods associated with an exclusively biomedical view of disease. An understanding of these phenomena can only be acquired through interpretations of the traces left by patients through language, attitude and action.

*Mimesis II* indicates the configuration of the narrative, the act of creating the plot, whose coherent thread structures it in meaningful order. Within the context of nursing, patients’ narratives can be obtained through so-called narrative interviews, which permit them to report lived experiences, or as Ricoeur would put it, to configure the pre-understanding of *Mimesis I*. The function of the interviewer is to listen to patients’ narratives, instead of asking pre-formulated questions. It is the patients themselves, therefore, who select the events that are significant to them and put them into words, without interference from the interviewer which might influence the process of selection and structuring of their patient’s discourse. The transcription involves a subsequent very delicate step, since—as Ricoeur claims—the written text is not a simple extension of the spoken words, but a process of “emancipation of the text from the oral situation” (Ricoeur 1981, 109), so that the meaning of the text frees itself from the underlying intention of the author to constitute a world of its own. The task of the interpreter does not involve looking *behind* the text with a view to discovering the patient’s psychology, for example, as Schleiermacher’s hermeneutic method tried to do. It consists, rather, in bringing to light that surplus of meaning capable of triggering a vaster variety of readings.

The act of taking one’s distance from the original narrative situation, which occurs when speech is converted into writing and becomes autonomous, constitutes an opportunity capable of generating further meanings: “the world of the text can cause the author’s world to explode” (Ricoeur 1981, 109). Not only does this objectification not go against interpretation, but, on the contrary, it provides a condition of possibility: the greater the distancing during the configurative phase, the greater the refigurative power of the text leading to *Mimesis III* (Charalambous et al. 2008, 638–639). *Refiguration*, according to Ricoeur, is “the intersection of the world of the text and that of the listener or reader” (1984, 77; 1998, 83): marks the accomplishment of the interpretative process, the moment when something occurs within the person reading the text turning him/her into a reader of his/her own self: the transformation of the living experience by effect of the text. The two dimensions of the hermeneutic arc refer, therefore, to each other: explaining more for a better understanding. This way, the explanation becomes the dialectical counterpart of understanding in the interpretational process, seen as a reciprocal, two-way spiral movement.

Research in the nursing field sees this hermeneutical model a tool by which to overcome the bottlenecks of an exclusively biomedical view of disease and treatment, which lies at the origin of a goodly number of ethical problems regarding the practice of the profession. It is also the outcome of a certain intolerance on the part of biomedical research based on the predominant organisational structure of articles published in scientific journals and known as IMRAD (Introduction, Methodology, Results, and Discussion), which, although designed to report the results of research in a concise and logically rigorous manner, appears insufficient when it comes to adequate exploration not only of what experiences of illness and suffering mean to patients, but also of the discomfort experienced frequently by nurses in the face of ethically problematic clinical situations (Lindseth and Norberg 2004, 149–151).

Therefore, the *Three Mimesis* inform three levels of reading—naïve reading, structural analysis, and comprehensive interpretation (Lindseth and Norberg 2004, 149–151)—that the literature of nursing has applied to two fields of qualitative research: on the one hand, within the context of better relationships with patients, when the interviews concern the experiences of diagnostic and therapeutic settings, on the other, self-understanding and critical reflection regarding the ethics of their profession, when it comes to interviews administered to nurses.

The objective of naïve reading is to obtain an initial understanding and recognise the general meaning of the text as a whole, in an effort to shift from a natural to a phenomenological attitude (Dan Zahavi and Martiny 2019, 161). The approach to the text—which has in any case already been fixed in the written form and therefore distanced from both the narrator and the reader—is open to register the immediate impressions of the health worker or researcher. Moreover, the process of identifying and transforming the key concepts of the text also presupposes conjectural elements. For example, the naïve reading of the experiences of a group of patients involved in cardiac rehabilitation revealed that the atmosphere during treatment had had a positive impact upon the patients; this was denoted not only through the narratives as such, but also thanks to the expressions of the patients observed in context (Simonÿ et al. 2018, 5).

Using structural analysis, a given test is divided into units of meaning, in an attempt to identify and formulate the topics it contains, which correspond to what was said by the patients and, according to some authors, to what the health-care professionals observed as well.^7^ During this phase, the narrative is subjected to distancing and acquires objective content, which goes beyond naïve understanding (Lindseth and Norberg 2004, 150; Pedersen et al. 2009, 8 and ff). Experiences are described not through abstract concepts, but condensed into phrases (e.g., “being at ease”, “feeling assisted”, “experiencing distress”, etc.). If a unit of language contains more than one essential meaning, it is divided further into sub-themes, which are compared with naïve understanding to validate it. If the structural analysis invalidates the naïve reading, the entire text is reread to achieve a new one, subsequently validated through a new structural analysis: this process is repeated until the naive understanding is validated effectively by recourse to structural analysis.

During the critical-interpretation phase, the aim is to understand the meaning of the text, read it again as a whole to formulate an understanding capable of revealing new significant aspects of phenomena previously taken for granted. Here it is the world of life that unfolds. Prefigured by the interviewees, configured in the interview and reconfigured in the interpretation of the researcher. This third level, *reconfiguration*, transforms the interview data into an opportunity for healthcare professionals to question their own clinical practice in order to be better able to grasp the responses provided by patients and gain a critical understanding of the ethical dimension of healthcare.

## From the interview to the world of the patient: some applications of the Ricoeurian method

Wiklund applies this three-level model to ways of understanding the phenomenon of suffering associated with a sense of the dignity of the sick, in order to come up with indications useful to guide medical and nursing staff regarding assistance modalities. The purpose of his study is to gain a deeper understanding of suffering and develop a theoretical model for relationships with patients. To achieve this goal, the Ricoeurian process of distancing is complemented by further reflection, where interpretations should be referred back to the empirical context (Wiklund et al. 2002, 114–125). Therefore, the colloquial, that is, the interactive interviews, carried out within two different contexts, between people with problems of drug addiction and between people operated recently on the heart, are transcribed to obtain as many narratives as possible regarding the lives and sufferings of the interviewees. The data obtained through the process of interpretation are subsequently exemplified using a single case, which was then discussed by extending it to a broader investigation of suffering. The results of the study showed how suffering is perceived as a painful effort, a clash between shame and dignity, while seeking affirmation and the right to be considered precious and unique. From here, doctors and nurses are able to draw conclusions regarding the ethics of care which prompted them to adopt attitudes favouring the protection and restoration of the dignity of their patients.

The phenomenological-hermeneutic model was also applied to the experience of feeling at home, as perceived over a long life span, by people between the ages of 2 and 102 (Zingmark et al. 1995, 47–60). From the data obtained from the narratives, it emerged that the perception of feeling at home constitutes an experience of considerable complexity, where feelings such as a sense of safety, rootedness, harmony, joy, privacy, togetherness, recognition, order, control, possession, nourishment, initiative, power and freedom all converge. The fundamental core is, in any case, a feeling of relationship with other significant people, in a significant place, carrying out significant activities. The understanding of this experience may be useful when planning the care of moderately and severely demented patients or of elderly residents in nursing-homes, in order to create a home-like environment.

It is interesting to notice that, at the first and second reading levels of the narratives of elderly residents in nursing homes or dying people, in order to be able to express the meaning of lived experience, the results are formulated not through the abstract terminology of scientific language (for example, solitude), but using the verbal expressions of everyday language (feeling alone) and by intercepting the recourse the narrators have to poetic expressions and metaphors (Norberg et al. 2001, 545–553). One way of achieving a deeper understanding of the world of the text, that is, the world of human suffering, is to seek the profound structures conveyed through metaphor. From this point of view, too, the reference is to Ricoeur’s “metaphoric turn”, that is, how metaphors expand the semantic dimension of ordinary speech, overcoming the resistance of habitual use of words and giving rise to a new, deeper understanding of phenomena (Ricoeur 1984, 6–8; 1978, 255 and ff). As a result, if patients’ metaphorical language cannot be deciphered, their experiences remain impenetrable and they themselves remain trapped inside their estrangement and distance from others. Not only will they experience further suffering but will stick to “safe” strategies to avoid revealing their true feelings in order to preserve a sense of dignity and avoid shame. For this reason, it is essential that nursing staff perform an almost “obstetric” function, by helping patients to “give birth” to what they really feel, in terms of discomfort and suffering (Wiklund et al. 2002, 121–122).

Also as regards the perception of the experience of ageing and old age the Ricoeurian method of reading and interpreting narratives is effective (De Juan Pardo et al. 2018, 9–17). The aim, once again, is to demonstrate the complexity of what, under the exclusive lens of the biomedical approach, would look like a simple organic phenomenon accompanied by a number of psychological implications. In this case, too, understanding is the outcome of the combination of different strategies availed of to ensure the reliability of the approach: from the interview phase until the final phase, where the results of the initial reading were correlated with those of the structural analysis and contrasted with literature on the subject, completes the hermeneutic arc, between explaining (using a rigorous and systematic scientific method) and understanding possible meanings of this phase of life (using an inductive method).

A structural analysis reveals interesting indications for an ethic of care of the elderly. First, the dichotomy between “getting old” and “feeling old” highlights how difficult it is to rigidly classify the category of the elderly on the sole basis of chronology; for some, the perception of old age is a goal to be reached, for others it means awareness of age-related changes and limitations with a consequent reduction of autonomy and fear of increasing dependence on others. Finally, a feeling of loneliness emphasises the need to provide the elderly with assistance that pays greater attention to remembering and telling and cares for it (De Juan Pardo et al. 2018, 9–17).

The reading and discussion of the results with a group of operators provided food for thought concerning an ethos of care of the elderly which paid greater attentive to the hermeneutics of the “symptom of ageing” rather than simply treating it. For example, the narratives highlighted the fact that care of the elderly becomes problematic when the answer to the question “who are the elderly?” is clothed in stereotypes and prejudices that cause the elderly to be marginalised. It emerged that the concepts of health and illness require clarification when applied to the elderly, because old age is not necessarily synonymous with illness, inactivity, dependence; on the other hand, the health of the elderly is not characterised by total absence of disorders. The result was awareness of the fact that taking care of the elderly requires attention not simply to their health, but also to their housing and relational needs, which can only be satisfied within an organic framework of services which enhances the network of family and friends created by the elderly during their entire lifetimes.

## Concluding remarks

The *Three Mimesis* and their levels of interpretation have been transposed and adapted to suit qualitative surveys of the health sector, with some leeway, at times, with respect to the model. What is certain is that, by electing the French philosopher as a source of inspiration, we obtain not only a method, but a different type of rationality which leads to a new paradigm of the art of healing. The "interpretive-phenomenology" approach can lead, on the one hand, to the compilation of medical history seen not simply as a "historia morbi", but also as a "historia aegri", that is, a revelation of the patient's existential world, which is not only symptomatic, but symbolic too (Svenaeus 2018). This can mean correction of any kind of possible “testimonial injustice” which might lead to non-acknowledgement of the patient's narrative as reliable and useful for diagnosis and therapy. On the other hand, the third level of Mimesis, *refiguration*, can shift the focus of the interview from the patient to the operator, favouring the interviewer’s interpretation of themselves and a critical examination of the ethical quality of the care they provide.

From the logic of efficiency and productivity, which related patients as a generality to standardised processes, the hermeneutic attitude advanced towards a complex world, where narration reveals suffering as the emblematic topos of singularity (Ricoeur 2007; Hettema 2014). This is why it is desirable that this exercise of "hermeneutical intelligence" be extended to all healthcare personnel, especially to hospitals and care of the elderly.

From this perspective stems the responsibility to act, which appeals to the practical wisdom of the health-care provider, for whom the dialectic between explaining and understanding becomes a tool for reconciling the universal—disease as a nosographic entity and treatment protocols—with details regarding the single patient, who experiences illness in a completely personal way.

## Footnotes

1 For this reason, some scholars propose supporting EBM with MBE, meaning Medicine Based (personalised) Evidence, aimed at guiding decision making for individual patients by profiling their clinical features (biology), values and life experiences (biographies). (Horowitz et al. 2017, 1246–1250).

2 “In the Fordist model of medicine, there must be a specific illness to treat; the evaluation of a doctor’s performance will then be made by counting the time required to treat as many livers as possible and the number of livers that get well”. Sennett 2008, 49.

3 The introduction of the electronic medical record which aimed at making patient data readily available, requires asking and answering a series of coded questions in a given time. This led to the practical inability of acquiring information useful to the creation of a complete picture of a given situation. Hartzband and Groopman (2016, 106–108) report the case of a woman who wrote down some questions she needed to ask the doctor during a follow-up visit, but was immediately blocked because, in order to consider issues outside the standard list, she had to schedule a special visit to discuss her concerns.

4 Benner (1994) considers the terms “interpretive” and “hermeneutical” interchangeable, though she retains the former more accessible than the latter.

5 This position, inspired by the thinking of Lévinas, presents, nevertheless, a theoretical and practical criticality. From a theoretical point of view, as Ricoeur himself observed (1992, 337–338), when discussing Lévinas, the priority of ethics over ontology might lead to a “hyperbole of otherness”, where responsibility for care would constitute an obligation without the possibility of creating a reciprocal relationship. From a practical point of view, this position, although driven by the more than justified intention of enhancing the ethical depth of care, risks de-professionalising nursing care.

6 “By social imaginary, I mean something far broader and deeper than the intellectual schemes people may entertain when they think about social reality in a disengaged mode. I am thinking, rather, of the ways people imagine their social existence, how they fit together with others, how things go on between them and their fellows, the expectations that are normally met, and the deeper normative notions and images that underlie these expectations” (Taylor 2004, 23).

7 According to most authors, the integration of patient interviews with practitioners’ observations constitutes a dynamic process between what is expressed verbally and what is observed through behaviour, body language and in the environment to provide a greater wealth of data and, therefore, a better understanding of the reality. (Simonÿ et al. 2018, 3–4).
